# Protein and the Adaptive Response With Endurance Training: Wishful Thinking or a Competitive Edge?

**DOI:** 10.3389/fphys.2018.00598

**Published:** 2018-05-23

**Authors:** Pim Knuiman, Maria T. E. Hopman, Conor Verbruggen, Marco Mensink

**Affiliations:** ^1^Division of Human Nutrition, Wageningen University and Research, Wageningen, Netherlands; ^2^Department of Physiology, Radboud University Medical Centre, Nijmegen, Netherlands

**Keywords:** dietary protein, endurance training, muscle adaptation, mitochondria, supplementation

## Abstract

The significance of carbohydrates for endurance training has been well established, whereas the role of protein and the adaptive response with endurance training is unclear. Therefore, the aim of this perspective is to discuss the current evidence on the role of dietary protein and the adaptive response with endurance training. On a metabolic level, a single bout of endurance training stimulates the oxidation of several amino acids. Although the amount of amino acids as part of total energy expenditure during exercise is relatively low compared to other substrates (e.g., carbohydrates and fat), it may depress the rates of skeletal muscle protein synthesis, and thereby have a negative effect on training adaptation. A low supply of amino acids relative to that of carbohydrates may also have negative effects on the synthesis of capillaries, synthesis and turn-over of mitochondrial proteins and proteins involved in oxygen transport including hamoglobin and myoglobin. Thus far, the scientific evidence demonstrating the significance of dietary protein is mainly derived from research with resistance exercise training regimes. This is not surprising since the general paradigm states that endurance training has insignificant effects on skeletal muscle growth. This could have resulted in an underappreciation of the role of dietary protein for the endurance athlete. To conclude, evidence of the role of protein on endurance training adaptations and performance remains scarce and is mainly derived from acute exercise studies. Therefore, future human intervention studies must unravel whether dietary protein is truly capable of augmenting endurance training adaptations and ultimately performance.

## Introduction

Nutritional strategies to maximize recovery from exercise are widely used by recreational as well as elite athletes. Post-exercise carbohydrate ingestion is considered to facilitate muscle glycogen resynthesis (Burke et al., 2017), and that of proteins to repair the exercise-induced damage to the contractile proteins and for *de novo* synthesis of proteins (Phillips, 2012). Thus far, scientific evidence demonstrating the significance of dietary protein is mainly derived from research with resistance exercise training regimes (Cermak et al., 2012). This is not surprising since the general paradigm states that endurance training has insignificant effects on skeletal muscle growth. This could have resulted in an underappreciation of the role of dietary protein for the endurance athlete.

**Figure 1:**
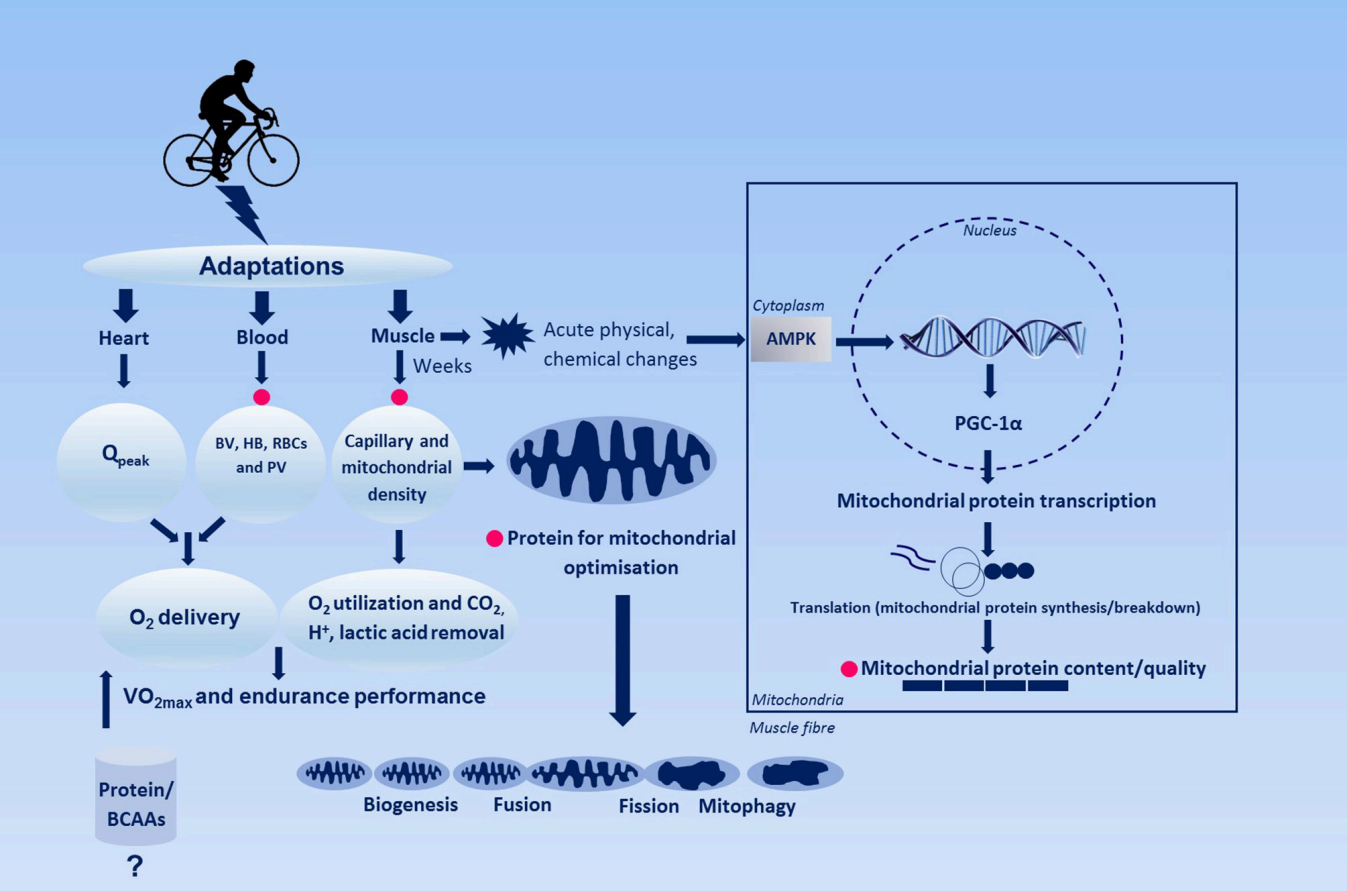
Simplified schematic figure representing the regulation of endurance training-induced adaptation and the hypothetical role of dietary protein herein. Changes in heart such as maximal cardiac output and blood, e.g., blood and plasma volume, hemoglobin, red blood cell volume enhances O_2_ oxygen-carrying capacity of the blood, whereas skeletal muscle adaptations potentially contribute to O_2_ extraction. Pink circles indicates where dietary proteins might play a role in adaptation with endurance training. AMPK, 5′adenosine monophosphate-activated protein kinase; BV, blood volume; HB, hemoglobin; PGC-1α, peroxisome-activated receptor gamma coactivator 1-alpha; PV, plasma volume; Q_peak_, peak cardiac output; RBCs, red blood cells.

However, a recent review on endurance training and skeletal muscle hypertrophy revealed that both acute and chronic endurance training enhances muscle protein synthesis and skeletal muscle growth respectively (Konopka and Harber, 2014). Particularly, they reported that eight out of nine studies on the effects of endurance training on skeletal muscle demonstrated significant muscle growth in both younger and older individuals (Konopka and Harber, 2014). Further evidence for increased protein-needs of individuals participating in endurance training regimes comes from studies on amino acid oxidation during exercise in rodents (Kato et al., 2016) and humans (Kato et al., 2016) and hypothetically for capillarization, synthesis and turn-over of mitochondrial proteins and proteins involved in oxygen transport including hemoglobin and myoglobin. Just as with resistance exercise, exogenous essential amino acids are required to repair the endurance exercise-induced muscle damage. From these considerations it becomes clear that the role of dietary protein in optimizing endurance training adaptations requires further study. Therefore, the aim of this perspective is to discuss the current evidence on the role of dietary protein and the adaptive response (e.g., biochemical and physiological endpoints) with endurance training. In addition, since the mechanisms underpinning these adaptations are not fully understood, we propose a novel hypothesis (Figure 1) based on our unpublished observations and the current literature why protein intake may potentially be advantageous for individuals participating in endurance training regimes.

## Adaptation to endurance training

Endurance exercise performance roughly depends on three major aspects: (I) maximal oxygen consumption (VO_2max_), (II) the percentage of VO_2max_ that can be sustained during endurance exercise, which in turn is largely dictated by the lactate threshold, and (III) mechanical efficiency, defined as the energy cost to sustain a power output or velocity (Wackerhage, 2014). Training these limiting factors may lead to an increase in oxidative capacity through hematological and metabolic adaptations and associated enhanced O_2_ transport and utilization (Montero et al., 2015). O_2_ transport is primarily regulated via cardiac (e.g., stroke volume) and circulatory (e.g., blood volume, O_2_ carrying capacity) adaptations, while improvements in O_2_ diffusion (involving capillaries) and utilization (involving mitochondria) are mainly the result of adaptations within skeletal muscle (e.g., mitochondrial capacity, capillary density, myoglobin, oxidative enzymes/proteins).

## Protein requirements for the endurance athlete

Synthesis and degradation rates of skeletal muscle proteins are usually in balance, ensuring that the amount of skeletal muscle proteins remains unchanged in healthy individuals (Burd et al., 2009). However, acute changes in different intramuscular protein fractions (mitochondrial, myofibrillar, sarcolemma) could be training specific. It has been proposed that endurance training augments the concentration of mitochondrial proteins without any changes in muscle size (Holloszy and Booth, 1976). Based on this idea, one could expect that different types of training would stimulate the intramuscular protein fractions differently. However, various authors reported similar increases of the effect of different exercise modes (endurance, resistance or concurrent) on mitochondrial protein synthesis during the early post-exercise period (Wilkinson et al., 2008; Robinson et al., 2010; Breen et al., 2011; Coffey et al., 2011; Burd et al., 2012; Donges et al., 2012). An increased need for dietary protein could partly arise from enhanced amino acid oxidation during endurance training (Tarnopolsky, 2004; Moore et al., 2014). Indeed, earlier studies have demonstrated increased amino acid oxidation rates through stimulation of protein breakdown rates (Lemon and Mullin, 1980; Bowtell et al., 2000; Harber et al., 2010; Howarth et al., 2010). Additionally, it has been theorized that endurance training affects amino acid requirements for an increased need of enzymes, for capillarization, and for hemoglobin and myoglobin synthesis (Tarnopolsky, 2004). The current sport science consensus statements on nutrition and athletic performance advises 1.2–1.4 g protein·kg^−1^·d^−1^ for endurance-trained athletes (American Dietetic Association et al., 2009; Jäger et al., 2017). However, Kato et al. (2016) studied the average protein requirements in endurance athletes during an acute 3-day training period using the indicator amino acid oxidation method and reported a recommended protein intake that is greater than the RDA (0.8 g · kg^1^·d^1^) and the current recommendations for endurance athletes (1.2–1.4 g·kg^−1^·d^−1^) (Kato et al., 2016). Moreover, they reported an estimated average requirement and a recommended protein intake of 1.6 of 1.8 g protein·kg^−1^·d^−1^ respectively. Therefore, it remains to be established whether these recommendations are optimal for individuals participating in endurance training regimes and whether this is affected by the training phase of the individual and other training parameters including intensity, type and frequency.

## Significance of protein ingestion before and during endurance training

Only a few investigations have addressed the role of protein ingestion before and during endurance training modalities. In line with previous work on protein ingestion prior to and during resistance exercise (Tipton et al., 2007), Coffey and colleagues reported that protein ingestion before a single bout of repeated sprints increases post-exercise myofibrillar protein synthesis (Coffey et al., 2011). However, short high-intensity endurance bursts such as repeated sprints differ both energetically and metabolically from prolonged continuous endurance training. For example, prolonged endurance training stimulates the oxidation of amino acids, in particular isoleucine, leucine and valine, otherwise known as the branched chain amino acids (BCAAs). Even though mitochondria are capable of oxidizing a variety of amino acids, they preferentially oxidize BCAAs (Phillips et al., 1993; Lamont et al., 1999; McKenzie et al., 2000). These amino acids including BCAAs can be used as a substitute for carbohydrates and fat as fuel source for ATP resynthesis. When compared to carbohydrates and fat, leucine oxidation during endurance exercise is relatively low (Lamont et al., 1990; Phillips et al., 1993; McKenzie et al., 2000; Tarnopolsky, 2004), yet, the absolute leucine oxidation increases because of the increase in total energy demand during endurance exercise. Since leucine is an essential amino acid and considered to be important for its role in translational machinery (Churchward-Venne et al., 2012), increased oxidation rates could depress the rates of skeletal muscle protein synthesis (Rose and Richter, 2009) and negatively affect protein requirements (Tarnopolsky, 2004). Other work by Koopman and colleagues demonstrated that the combined ingestion of protein and carbohydrate throughout a prolonged endurance exercise bout (2.5 h cycling, 1 h of running and 2.5 h of cycling) improves whole body net protein balance at rest, as well as during exercise and post-exercise (Koopman et al., 2004). The enhanced whole body net protein balance with protein ingestion may be partly explained by the diminished muscle protein breakdown during endurance exercise (Hulston et al., 2011). Furthermore, the favored enhanced net protein balance has been proposed as the theoretical basis for a potential ergogenic effect of protein ingestion during endurance exercise. Yet, findings of combined carbohydrate and protein ingestion during endurance exercise on performance outcomes are controversial (Saunders, 2007). For instance, in a study by Saunders et al. (2009), male cyclists performed two 60 km time trials with either carbohydrate or carbohydrate + protein beverage every 5 km (200 ml) and post-exercise (500 ml). No significant difference in 60 km total time between the conditions was found. In spite of that, the addition of protein hydrolysate to the carbohydrate beverage explained a significant amount of variance in performance times between conditions during the final stages (20 and 5 km) of the time trial (Saunders et al., 2009). The latter suggests a favorable effect of protein ingestion during exercise on endurance performance. Lastly, the addition of protein to a carbohydrate supplement consumed during exercise does not improve recovery or performance in elite cyclists despite high demands of daily exhaustive sessions during a 1-week training camp (Hansen et al., 2016). In summary, there is currently little evidence for improved endurance performance with protein intake before and during endurance exercise. Nevertheless, findings of abovementioned studies underline the importance of exogenous protein for remodeling/repair of the exercise-induced damaged protein.

## Significance of protein ingestion after endurance exercise

Since most of the research so far has focused on protein ingestion with resistance exercise, these findings form generally the basis for protein ingestion recommendations for individuals participating in endurance-based programs. However, the skeletal muscle adaptive response during post-exercise recovery is strongly affected by food intake. Post-exercise supplementation in the form of protein after exercise has been the focus of many acute exercise interventions (Howarth et al., 2009; Harber et al., 2010; Coffey et al., 2011; Lunn et al., 2012). Only a few studies have examined the responses of dietary protein on mitochondrial protein synthesis after endurance exercise. Breen et al. (2011) examined the role of dietary protein on both mitochondrial and myofibrillar protein synthesis (Breen et al., 2011). In their study, trained healthy males cycled for 90 min at ~77% VO_2max_. Such intensity (~77% VO_2max_) can be considered as vigorous endurance exercise (Garber et al., 2011) and results in more mitochondrial mass and improved skeletal muscle oxidative capacity when applied chronically. Immediately and 30 min following the exercise bout, subjects ingested a carbohydrate beverage and in one condition a total of 20 g of whey protein was added. It was shown that the co-ingestion of whey protein with carbohydrate augments the myofibrillar protein synthetic response up to 4 h after exercise (Breen et al., 2011). Their finding, namely that endurance exercise with post-exercise dietary protein ingestion enhances myofibrillar protein synthesis, is in accordance with previous findings where subjects ingested protein after high-intensity sprint exercise (Coffey et al., 2011). Noteworthy, the authors did not find a difference between the conditions on mitochondrial protein synthesis. It might be possible that the timing of the biopsy overlooked any potential increase in mitochondrial protein synthesis. Indeed, recent work by Hill et al. (2013) demonstrated that the acute PGC-1α mRNA, considered as the master regulator of mitochondrial biogenesis, was enhanced after a 60 min endurance bout (~70% VO_2max_) at 6 h when subjects were exposed to a 2-week dietary intervention with co-ingestion of carbohydrates + whey protein isolate (Hill et al., 2013). Their finding that carbohydrates + whey protein isolate enhanced a marker of mitochondrial recovery is in contrast with the findings of Breen et al. (2011). The different findings are possibly explained by the timing of the muscle biopsies and the applied nutritional strategy. For example, subjects in the study of Hill and colleagues were supplemented for 2 weeks with either carbohydrates or carbohydrates + whey protein isolate (Hill et al., 2013), whereas subjects in the study of Breen et al. (2011) were merely supplemented post-exercise (Breen et al., 2011). Lastly, even though mitochondrial protein synthesis and PGC-1α mRNA can be both used as a marker for mitochondrial recovery, comparison of findings remains difficult.

## Significance of protein ingestion during the prolonged recovery period after endurance exercise

At this moment, there is not much research on the effects of protein ingestion during the prolonged recovery period after endurance exercise (from 3 up to 12 h post-exercise). However, Areta et al. (2013) reported that the distribution of protein ingestion during the 12 h after resistance exercise affects the rates of myofibrillar protein synthesis (Areta et al., 2013). Specifically, in their study they compared three isocaloric timing strategies for protein ingestion during a 12 h period after resistance exercise: (I) 2 × 40 g every 6 h (bolus); (II) 4 × 20 g every 3 h (intermediate); and (III) 8 × 10 g every 1.5 h (pulse). It was concluded that intermediate feeding was superior to either bolus or pulse feeding for stimulation of myofibrillar protein synthesis. Albeit somewhat speculative, it is likely that individuals participating in an endurance training regime also benefit from an intermediate protein ingestion strategy. Further on this notion, work from Breen et al. (2011) showed that the addition of protein to a carbohydrate drink in the early post-endurance exercise did not show increases in mitochondrial protein synthesis compared with a carbohydrate drink only (Breen et al., 2011). However, mitochondrial protein synthesis measurements were taken in the early post-exercise endurance period, since the latency of mitochondrial protein synthesis is currently unclear, it could be that a response at a later stage was overlooked.

## Long-term endurance training and the role of protein

Unfortunately, to the best of our knowledge, the current literature still lacks studies exploring the role of dietary protein with a robust period of endurance training (>6 weeks). The period of the endurance training intervention is an important aspect when looking at how skeletal muscle adaptations can be influenced by dietary protein. Indeed, recent work by Montero et al. (2015) demonstrated that the increase in VO_2peak_ with 6 weeks of endurance training (3–4 endurance sessions per week at ~65% W_max_) was primarily explained by an increase in peak cardiac output and oxygen-carrying capacity of the blood (Montero et al., 2015). Moreover, in their study, skeletal muscle adaptations related to muscle capillarization and mitochondrial volume density did not substantially contribute to the improvements in VO_2peak_ following the 6 weeks of endurance training. It is therefore important to conduct endurance training intervention studies over a longer period (e.g., 8, 12 or 20 weeks), especially when the purpose of the study is to explore the role of protein within the entire spectrum of the physiological adaptive response to endurance training. Only a few studies investigated the effects of protein supplementation during “long-term” (4–6 weeks) endurance training in healthy young men (Ferguson-Stegall et al., 2011) and older adults (Robinson et al., 2011). In the study of Robinson et al. (2011) young and old participants performed 3 treadmill-based aerobic sessions weekly for 6 weeks (30 min; intensity increased progressively ranging from ~65 to 85% HR_max_) and were provided with a post-exercise beverage containing either carbohydrates or isocaloric protein. The absolute VO_2max_ increased in the protein group but not in the carbohydrate group following 6 weeks of aerobic training (Robinson et al., 2011). The finding that protein supplementation improves endurance training-induced oxidative adaptations is supported by the study of Ferguson-Stegall et al. (2011). In this study it was demonstrated that 60 min of cycling, five times a week (~75–80% VO_2max_) for 4 weeks with intake of a post-exercise chocolate milk beverage, improves VO_2max_ and body composition to a greater extent compared to carbohydrates alone. Somewhat surprisingly, markers of mitochondrial adaptation such as citrate synthase activity, succinate dehydrogenase activity and PGC-1α increased as a result of training independent of the type of nutritional intervention (Ferguson-Stegall et al., 2011). Finally, supplementation of a mixture BCAAs in mice increased mitochondrial biogenesis and whole body physical endurance as measured as the time till exhaustion in a treadmill test (D'Antona et al., 2010). Since sport specific performance outcomes were not included in aforementioned studies, it remains unclear whether the increase in VO_2max_ improved the performance.

## The role of protein source

The superiority of one protein source over another in terms of exercise adaptation has not been convincingly demonstrated (Campbell et al., 2007), though type and quality can influence bioavailability (Jäger et al., 2017). The choice of protein source is of additional relevance to athletes given the environmental/ethical, overall health, and bioactivity differences which have been reported (Grunert et al., 2000; Tang et al., 2009; Song et al., 2016; Giromini et al., 2017). In the context of this perspective, it is the bioactivity of different protein sources that is of primary consideration, given the potential beneficial effects on oxygen diffusion and utilization. The “food first” approach to sports nutrition is widely touted, but food structure can influence the suitability of protein sources via altered kinetics of amino acid availability (Dangin et al., 2002; Fardet et al., 2018) and digestive discomfort (de Oliveira et al., 2014). For these reasons as well as convenience, powder-form supplements are often recommended. Given the further geo-logistic, time, and dosage demands of endurance sport, tablet-form protein supplements may be of greater facility in some instances. On a metabolic level, the presence or absence of essential amino acids (Hoffman and Falvo, 2004), leucine content (Norton et al., 2010), and type of protein (Tang et al., 2009) have been shown to alter the muscle protein synthetic response and body compositional changes, prompting the development of a number of assessment methods. Despite a range of assessment scales existing (Hoffman and Falvo, 2004; Jäger et al., 2017), comparisons of protein sources have centered largely on biological value (proportion of nitrogen used for tissue formation) and digestion rate (Dangin et al., 2002; Bauer et al., 2013). Multiple authors have reported differences in biological value between protein sources (Hoffman and Falvo, 2004; Bauer et al., 2013). In addition, it has been demonstrated that more rapidly digested sources (i.e., whey) seem to confer increased insulin response, post-exercise muscle protein synthesis, resting muscle protein synthesis and blood leucine enrichment in the first hour after consumption (Tang et al., 2009). However, the suitability of the existing assessment scales varies with respect to their relevance to endurance athletes (as does the use of an inappropriately short 1-h assessment window). For instance, the Protein Efficiency Ratio (PER), representing mass gain per g of protein ingested, represents an inverse of suitability for most endurance athletes. Biological value too may be of limited use to endurance athletes as it considers only the tissue-related nitrogen use (thus omitting protein synthesis of oxidative enzymes and hematopoiesis, for instance). The Protein Digestibility Corrected Amino Acids Score (PDCAAS) is the most widely used assessment, but it too is limited by its lack of consideration of ileal digestibility, and the short-sightedness of determining protein quality based on the content of a single amino acid (which may be sufficiently abundant in the habitual diet). Endurance athletes demonstrate prolonged periods of increased muscle protein synthesis while still engaged in exercise, meaning protein requirements are elevated while in a state of compromised gastric function and reduced feeding opportunities (van Wijck et al., 2012). The PDCAAS may be of assistance to athletes engaging in ultra-endurance events, by informing a decision which should maximize essential amino acids per weight consumed, to minimize the risk of gastrointestinal distress. Coupled with personal experience of irritability triggers, this approach may be particularly practical for ultra-endurance runners and triathletes (events >3 h duration).

### Amino acid profile bioavailability

There is a lack of understanding concerning the practical relevance of established differences in source bioactivity on the adaptive response to endurance training, though some reports when combined suggest an adaptive advantage (Tang et al., 2009; Konopka and Harber, 2014). Unpublished data from our own study of supplementary effects of proteins on endurance training adaptation showed a response effect of habitual carbohydrate intake, before controlling for the expected variation due to casein supplementation. This suggests that the adaptation effect of supplementing even a robust bioavailable protein source is relatively low in the context of dietary effects on training outcomes, and so the importance of protein source is somewhat diminished.

Wolfe in his 2000 article raised the suggestion that endurance athletes' optimal protein intake may seek to maximize recovery (protein synthesis) while avoiding weight gain (protein deposition) (Wolfe, 2000). With this goal, one may seek to provide an endurance-tailored amino acid profile which avoids a hypertrophy-oriented profile (e.g., high in leucine) and instead provides an amino acid profile more specific to the requirements of oxygen utilization. I.e., those necessary for mitochondrial biogenesis (PGCs), fission (Fiss1, MPP, Drp1), mitophagy (Pink1, Parkin), fusion (Opa1, Mfn1/2), and the production of rate-limiting mitochondrial enzymes (pyruvate dehydrogenase, carnitine palmitoyl transferase). The amino acid composition of the parenthesised drivers of these processes may serve as a start-point for the elucidation of an endurance-specific ideal in terms of amino acid profile. However, the profile of downstream products, endogenous amino acid bioavailability, and the quantities involved ought to be considered. The leucine-induced increase in muscle protein synthesis is contested in humans, and high quantities may decrease autophagy (a vital aspect of endurance-specific adaptation) (Glynn et al., 2010). As such, the mechanisms of leucine's effects on body composition, muscle protein synthesis initiation factors (e.g., 4E-BP1) and autophagic regulators (e.g., ULK1) should be shown to be preferential in an endurance setting before disproportionate inclusion in supplements.

### BCAAs, casein or whey

It has been reported that BCAAs are preferentially oxidized (ahead of un-branched amino acids) during endurance exercise (Hood and Terjung, 1990; MacLean et al., 1994). Given that BCAAs are also essential and that muscle protein synthesis is elevated following but also during exercise (Konopka et al., 2017), extended endurance exercise may evoke an environment of suboptimal BCAA availability. For this reason it may be beneficial to the overall adaptive response and/or performance (via substrate availability) to provide a source rich in BCAAs during extensive exercise (e.g., whey, soy or casein). When used concurrently with less intensive endurance training, high intensity and/or strength training will evoke a greater myofibrillar protein synthetic response (Egan and Zierath, 2013; Philp et al., 2015), which may improve performance adaptation (Howarth et al., 2009). The existing wisdom regarding protein supplementation is thus more likely to hold true; a readily digested source of protein with high leucine content (e.g., whey) may be preferable to maximize post-exercise muscle protein synthesis (Tang et al., 2009). In these situations protein supplementation may aid in satiety as well as achieving protein intake targets while total energy demands of training are likely decreased.

As described earlier in this paper, mitochondrial protein synthesis demonstrates a delayed response post-exercise when compared to myofibrillar protein synthesis (Di Donato et al., 2014; Philp et al., 2015). It then follows that the acute protein requirement for endurance athletes immediately following exercise may be reduced, while the window of elevated utilization may exist for a longer time-period compared to strength trainers. If true, the existing advice paradigm of rapidly absorbed protein to be ingested as soon as possible post-exercise may sub-optimally support the protein-synthetic adaptive response to endurance training. In consideration of this observation, protein with a slower digestion rate may be preferable. However, it has been suggested that essential amino acid content and rapid digestion tend to coexist in protein sources (Tang et al., 2009). Furthermore, the greater insulin response reported to accompany these properties, which may contribute to digestion rate, is likely unavoidable in the context of post-endurance exercise refueling of muscle glycogen. Consumption of post-exercise protein in a whole-food form including dietary fiber to prolong the period of elevated amino acid availability is one simple solution. Division of the dose between two meals may also be necessary in order to optimally provide for protein availability over the 6 + h of elevated mitochondrial/cytosolic protein synthesis.

## Conclusion

To summarize, evidence of the role of protein on endurance training adaptations and performance is scarce. Yet, a number of acute endurance exercise studies have raised interesting hypotheses. However, these hypotheses are mainly based on studies measuring muscle protein synthesis, physiological (e.g., amino acid oxidation) and biochemical (e.g., activation/phosphorylation of specific enzymes/proteins or mRNA profiles) endpoints which do not necessarily reflect an improved adaptation and performance. Even though the findings of acute exercise studies contribute to the understanding of the mechanisms that underpin adaptation with endurance training, it is no direct proof that individuals performing endurance training benefit from additional protein. Future evidence must be derived from long-term endurance training studies that combine performance outcomes and biochemical/physiological endpoints.

## Author contributions

PK wrote the perspective. MH, CV, and MM contributed substantially by giving insightful comments and suggestions during the creation of the perspective.

### Conflict of interest statement

The authors declare that the research was conducted in the absence of any commercial or financial relationships that could be construed as a potential conflict of interest.
